# Hemolysis, hemolytic markers, and mortality in sepsis: a scoping review

**DOI:** 10.1186/s40635-025-00786-0

**Published:** 2025-08-05

**Authors:** Victoria Bünger, Stephanie Scholz, Martin Russ, Steffen Weber-Carstens, Jan A. Graw

**Affiliations:** 1 Department of Anesthesiology and Intensive Care Medicine, CCM/CVK Charité-Universitätsmedizin Berlin, Corporate Member of Freie Universität Berlin, Humboldt-Universität Zu Berlin, Campus Virchow-Klinikum, Augustenburger Platz 1, 13353 Berlin, Germany; 2 ARDS/ECMO Centrum Charité, Charité-Universitätsmedizin Berlin, Berlin, Germany; 3 Department of Anesthesiology and Intensive Care Medicine, Universitätsklinikum Ulm, Ulm University, Ulm, Germany; 4 Department of Anesthesiology, Friedrich-Alexander-Universität Erlangen-Nürnberg (FAU), Universitätsklinikum Erlangen, Erlangen, Germany

**Keywords:** Hemolysis, Sepsis, Cell-free hemoglobin, Haptoglobin, Bilirubin

## Abstract

**Background:**

Despite advanced and early treatment in the intensive care unit (ICU), mortality in patients with sepsis remains high. Sepsis is often associated with hemolysis. In the clinical routine, hemolysis is often overlooked, as markers of hemolysis are often not routinely measured. Aim of this scoping literature review is to quantify the incidence and extent of hemolysis in patients with sepsis and the association with mortality.

**Methods:**

Systematic literature search in bibliographic databases MEDLINE, EMBASE and Web of Science for sepsis and hemolysis.

**Results:**

A total of 3382 studies underwent title-abstract screening and 169 studies were reviewed in full. There were 34 studies with a total of 27,702 patients with sepsis and reported hemolysis or hemolytic markers and clinical outcomes included in the final analyses. Mortality ranged from 5.4 to 78.6% with a mean mortality of 20.1% across all studies. A significant association between hemolysis or hemolytic markers with increased mortality was observed in nine studies.

**Conclusions:**

Although significant associations between hemolysis and outcome in patients with sepsis were observed, hemolytic markers are not yet routinely and regularly monitored in clinical routine on the ICU. Hemolytic markers can provide information about disease severity and outcome on ICU admission and during the course of the disease. Future work should focus on identification of reliable markers of hemolysis with a potential for easy and timely measurements ideally at the patients’ bedside. With an additional definition of monitoring standards, the potential of hemolysis monitoring for prognostication and therapeutic approaches will emerge.

**Graphical abstract:**

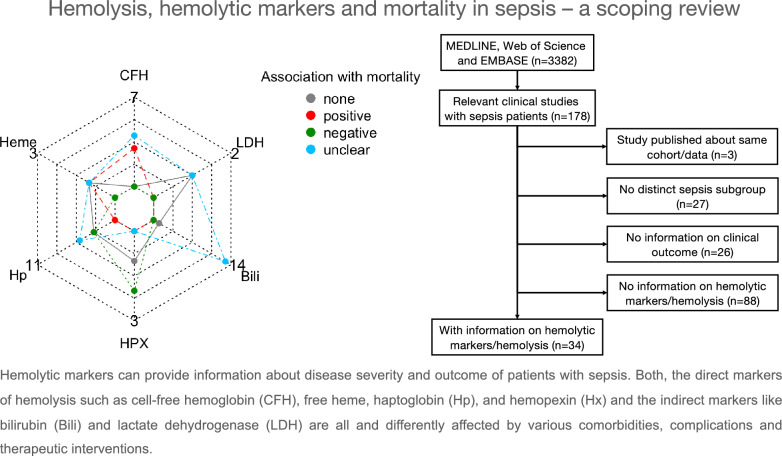

**Supplementary Information:**

The online version contains supplementary material available at 10.1186/s40635-025-00786-0.

## Background

Sepsis is a common, life-threatening condition accounting for approximately 20% of annual global deaths. Despite advanced and early treatment in the intensive care unit (ICU) mortality remains high, ranging up to 35% [1, 2]. Sepsis is often associated with additional complications such as end-organ injuries, shock, coagulopathies, and hemolysis [3, 4]. Hemolysis in sepsis is considered to be attributed to transfusion reactions and complement activation, disseminated intravascular coagulation, capillary stop-flow, restriction of glucose availability to red blood cells, changes in red blood cell membrane properties, hemolytic pathogens, and red blood cell apoptosis [5].

When red blood cells hemolyze within the blood stream, their contents such as cell-free hemoglobin (CFH), electrolytes like potassium, and enzymes like lactate dehydrogenase (LDH) are released into the vasculature. Furthermore, hemoglobin degradation products free heme and free iron are liberated [6]. CFH can bind nitric oxide (NO) with high affinity inducing consecutive vasoconstriction [7]. Furthermore, CFH acts proinflammatory and induces platelet aggregation [8]. Cell-free heme activates Toll-like receptor-4 (TLR-4) and proteasomes, causes lipid peroxidation and mitochondrial damage, and increases the production of reactive oxygen species. Furthermore, free iron promotes inflammation and is an important substrate for bacterial growth [9–11]. Mammals possess the hemoglobin-scavenger haptoglobin (Hp) that can bind CFH in an Hp–hemoglobin complex which is removed from plasma by CD-163 mediated endocytosis by hepatic and splenic macrophages [12, 13]. Hepatic derived glycoprotein hemopexin (Hx) binds free heme in an Hx–heme complex which is degraded in the liver [14]. Consequently, both scavenger systems are depleted in situations of increased hemolysis with a consecutive accumulation of CFH and free heme in the plasma [14].

Current data suggest a positive correlation between CFH and increased mortality in patients with sepsis [15, 16]. In addition, reduced plasma concentrations of Hp and Hx appear associated with an increased mortality in these patients [3, 16]. In patients with the acute respiratory distress syndrome (ARDS) and treatment with extracorporeal membrane oxygenation (ECMO), increased plasma concentrations of CFH and decreased plasma concentrations of Hp are associated with increased ICU-mortality rates and a higher incidence of renal failure [17–19].

In the clinical routine, hemolysis is often overlooked, because markers of hemolysis are frequently not measured routinely. The aim of this scoping literature review was to collect data on the incidence of hemolysis in ICU patients with sepsis and to determine a potential association of hemolysis with mortality and organ failure in septic patients.

## Methods

### Literature search strategy

A comprehensive literature search of clinical studies in septic patients reporting clinical outcomes and data on hemolysis was conducted on November 15, 2024, in three bibliographic databases (Pubmed MEDLINE, EMBASE/Ovid (1947–present) and Web of Science (1900–present); publication dates between 1932 to present using Preferred Reporting Items for Systematic Reviews and Meta-Analyses (PRISMA) guidelines. The following search terms were used: (plasma haemoglobin OR plasma hemoglobin OR cell-free haemoglobin OR cell-free hemoglobin OR free haemoglobin OR free hemoglobin OR haptoglobin OR haemolysis OR hemolysis) AND (Sepsis OR Septic shock OR Bacteremia OR Fungemia OR Septicemia OR Systemic inflammatory response syndrome OR SIRS). Details on the search strategy are available in Additional File 1. The study protocol was registered in advance on Open Science Framework on April 1, 2024 (10.17605/OSF.IO/S8YB3).

### Inclusion and exclusion criteria

All studies found by the above-mentioned search terms were included for further analysis. Preclinical studies (non-human and experimental studies), reviews, comments, editorials, case reports, guidelines and conference abstracts were excluded. Furthermore, studies on pediatric or pregnant patients were excluded. Included were studies published in (1) English or German on (2) adult and sepsis patients with (3) reported clinical outcomes (mortality) and (4) reported markers of hemolysis. Duplicates were removed automatically by the literature screening Software Rayyan and manually [20]. Title-abstract screening was performed independently by two reviewers (V.B., S.S. or J.A.G.) and conflicts were decided by a third reviewer by majority vote. If sufficient information was not derived from title and abstracts but the inclusion criteria were met, the articles were transferred for full-text screening, also conducted by two independent reviewers (V.B., S.S. or J.A.G.). Conflicts were then discussed by all three reviewers.

### Data extraction

The following data were extracted from the included eligible studies: year of publication, year and country of observation, number of patients, study population characteristics [age, sex, Sequential Organ Failure Assessment (SOFA) score, Acute Physiology And Chronic Health Evaluation (APACHE) score, etiology of sepsis, development of ARDS], mortality, definition and incidence of hemolysis and quantification of hemolytic markers. Data on transfusion of packed red blood cells (PRBCs), therapy with ECMO and extracorporeal therapies such as renal replacement therapy (RRT) were extracted, if available. Data were extracted and controlled by S.S. and V.B., and discrepancies were revisited by both authors.

## Results

**Fig. 1:**
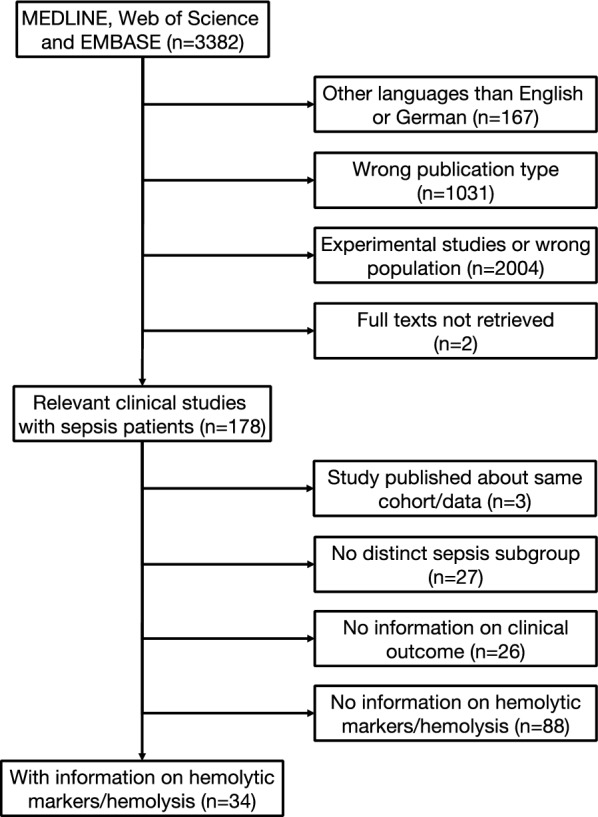
Flowchart for study inclusion

Details of the results of literature research and study inclusion are demonstrated in Fig. 1. There were 34 clinical studies with information on hemolysis published between 1971 and 2024 and relevant data were extracted (Table 1).

**Table 1:** Characteristics of included studies

Author Year	Place of study, study type and design	ICU	Patients with sepsis	Mortality in sepsis/all septic patients (%)	Age [median, 25%, 75%, median (range) or mean ± SD]	Gender, no. male (%)	SOFA-Score, time of assessment	Parameter for hemolysis	Endpoints
Adamzik 2012	Essen, Germany, single center, observational prospective	yes	161	30 days: 50/161 (31.1%)	survivors: 60 (48,72) non-survivors: 56 (49,66)	92/161 (57.1%)	Survivors: 11 (8.0,14.0), day 1 non-survivors: 12 (10.0,15.0), day 1	CFH	30-day survival
Boshuizen 2019	Amsterdam, NL, two centers, prospective observational cohort	yes	34	Hospital: 14/34 (41.2%)	64.5 (59,74)	13/34 (38.2%)	8 (5,9), study inclusion	Hp Free heme	Iron metabolism Hospital mortality
Caya 1986	Wisconsin, USA, two centers, retrospective	n/a	36	24/36 (66.7%)	45 (18–77 range)	24/36 (66.7%)	n/a	None	Analysis of clostridial sepsis in patients with leukemia*
Czempik 2023	Katowice, Poland, single center, prospective	yes	35	8/35 (22.9%)	69 (60,73)	20/35 (57.1%)	9 (7,12), n/a	Bili	Impact of intravenous iron on Ret-He and Ret subpopulations
Domizi 2024	Marche, Italy, single center, prospective observational	yes	50	Hospital: 8/50 (16%)	58 ± 16	40/50 (80%)	10 (7,11), ICU admission 9 (8,11), study inclusion	CFH	Hospital mortality perfused vessel density after acetaminophen, SOFA
Ekregbesi 2018	London, UK, single center, prospective observational	yes	70	Hospital: 19/70 (27.1%)	64 (18–89 range)	44/70 (62.9%)	7 (3–16 range), ICU admission	Free heme Hp HPX	In-hospital mortality, APACHE and Hp, Heme or HPX
Englert 2019	Jena, Germany, single center, prospective	yes	18	28 days: 5/18 (27.78%)	65.5 (56.25,75.25)	14/18 (77.78%)	11 (8,13), day 1	Free heme Bili	28-day mortality hypalbuminemia and labile heme in sepsis, SOFA and Heme/HSA
Fujita 2010	Tokyo, Japan, single center, retrospective	mixed	18	30 days: 5/18 (27%)	75 ± 2* (range 59–88)	13/18 (72.2%)	n/a	n/a	Clinical and epidemiological features of C. Perfringens sepsis
Gaini 2007	Odense, Denmark, single center, prospective, observational	n/a	74	28 days: 4/74 (5.4%)	62.6 ± 19.2	38/74 (51.35%)	2.1 ± 1.5, study inclusion	Bili	Describe levels of HMGB1 in non-critical ill patients suspected of having sepsis
Gaini 2008	Odense, Denmark, single center, prospective	n/a	86	28 days: 10/86 (11.6%)	67.1 ± 17.2	45/86 (52.3%)	2.9 ± 1.8, study inclusion	Bili	Mortality d28 Relation of HMGB1, sCD163 and PCT/LBT/IL-6/IL-10 with disease severity
Han 2015	Pennsylvania, USA, single center, prospective, observational	Yes	31	Hospital: 5/31 (16.1%)	60.8 ± 18.4	23/31 (74.2%)	n/a	Hp	In-Hospital mortality Negative predictive value
Janz_1 2013	Nashville, USA, single center, observational prospective	Yes	391	Hospital: 85/391 (21.7%)	57 (48,68)	218/391 (55.8%)	n/a	CFH	Hospital mortality
Janz_2 2013	Nashville, USA, single center, prospective, observational	Yes	387	Hospital: 84/387 (21.8%)	57 (48,68)	216/387 (55.8%)	n/a	Hp HPX CFH	Hospital mortality
Janz 2015	Nashville, USA, single center, prospective, interventional	Yes	40	Hospital: 5/40 (12.5%)	Acetaminophen (*n* = 18): 50 (41,64) Placebo (*n* = 22): 58 (47,63)	21/40 (52.5%)	Acetaminophen: 5.5 (4,8.5), study inclusion Placebo: 6 (5,8), study inclusion	CFH	In-Hospital mortality plasma-F2-IsoP on day 3
Kelly 2018	Pennsylvania, USA, single center, prospective, observational	Yes	139	14 days: 18/139 (12.9%) Hospital: 41/139 (29.5%)	62.1 (IQR 23.2)	51/139 (36.7%)	n/a	Hp	14-day and in-hospital mortality
Kerchberger 2019	Nashville, USA, single center, prospective, observational	Yes	496	Hospital: 106/496 (21.4%)	57.4 ± 15.6	265/496 (53.4%)	n/a	CFH Hp	Development of ARDS during sepsis
Kingston 2020	Chittagong, Bangladesh and Rourkela, India, two centers, prospective, observational	Mixed	56	Hospital: 14/56 (25%)	34 (25,51)	30/56 (54%)	n/a	Bili CFH	(Hospital mortality), SVRI, MAP, Vasoconstriction, tissue perfusion
Lan 2022	MIMIC-III, Boston, USA, Retrospective	Yes	501	28 days: 223/501 (44.5%) 90 days: 268/501 (53.5%) ICU: 199/501 (39.7%) Hospital: 228/501 (45.5%)	≥ 60 yr: 302 (60.3%)	267/501 (53.3%)	9 (7,12), ICU admission	Hp	28-day, 90-day, ICU and hospital mortality, SOFA
Larsen 2010	Rio de Janeiro, Brazil, two centers, observational prospective	Yes	52	28 days: 18/52 (34.6%) Hospital: 28/52 (54.0%)	69 (range 51–82)	28/52 (54%)	9 (range 6–11), day 1	HPX	28-day mortality SOFA-Score (day 1–7)***
Leff 1992	Colorado, USA, single center, prospective, observational	Yes	29	10/29 (34.5%)	57.2 ± 3.3	17/29 (58.62%)	n/a	Bili Hp	ARDS in septic patients (enzyme activity, biomarkers)
Maiden 2018	Victoria, Australia, multicenter retrospective (ARISE)	Mixed (85.6%)	1275	ICU: 109/1275 (8.5%) Hospital: 162/1275 (12.7%) 28 days: 163/1275 (12.8%) 90 days: 193/1275 (15.1%)	65 (51,75)	782/1275 (61.3%)	n/a	Bili	Patients with PRBCs within 72 h, Hb and volume administration during resuscitation from septic shock
Memis 2002	Edirne, Turkey, single center, prospective, RCT	Yes	30	8/30 (26.7%)	52.5 (41.0,68.3)	18/30 (60%)	6.6 ± 4.2, baseline 6.4 ± 4.1, post infusion 6.1 ± 3.7,24 h 6.2 ± 3.6,48 h	Bili	Cytokine level after methylene blue infusion
Mizuno 2022	Shiga University, Japan, Single center, retrospective	Yes	78	20/78 (25.6%)	73 (67,80)	23/55 (41.8%)	9 (6,12), Within first 24 h after admission	Hp	28 day mortality, Regression for 180 day mortality, cytokines in septic patients
Nilsson 2020	Lund, Sweden, single center, retrospective	Yes	474	90 days: 182/474 (38.4%) 180 days: 207/474 (43.7%)	65 ± 15	268/474 (56.5%)	Control: 8 (5,11), first SOFA RBC: 8 (5,10), first SOFA Control: 9 (7,12), max SOFA (within 28 days) RBC: 11 (8,13), Max SOFA (within 28 days)	Bili	Mortality, AKIN/RRT, circulatory and respiratory failure, SOFA-Score for liberal transfusion strategy
Reah 1997	Leeds, UK, single center, prospective, observational	Yes	14	28 days: 9/14 (64.3%) Overall: 11/14 (78.6%)	65 (51,71)	10/14 (71.4%)	n/a	Bili	Hemodynamic and toxicologic effects of diasporin cross-linked hemoglobin
Sharma 2019	Sao Paulo, Brazil, three centers, prospective, observational	Yes	27	19/27 (70.3%)	62.4 ± 12.8	19/27 (70.4%)	8.3 ± 2.9, n/a	Hp	Impairment of lipid metabolism in septic patients
Shindo 2015	Jichi, Japan, single center, retrospective	n/a	21	5/21 (23.81%)	76 (71,83)	10/21 (47.6%)	n/a	Bili LDH	Epidemiological and pathobiological profiles of C. Perfringens
Spies 1994	Prospective, single center RCT	Yes	58	35/58 (60.3%)	48.9 ± 15.4	44/58 (75.86%)	n/a	Bili	VO2, gastric mucosal pH and va CO2-gradient in septic shock
Staudinger 1996	Vienna, Austria, single center, prospective, interventional	Yes	24	7/24 (29.17%)	51.5 ± 16.5	16/24 (66.7%)	n/a	Hp	Influence of pentoxifylline on cytokines in patients with septic shock
Sun 2024	MIMIC-IV, Boston, USA retrospective	Yes	22,633	Hospital: 3451/22,633 (15.2%) 30 days: 4255/22,633 (18.8%)	65.0 ± 16.4	13,077/22,633 (57.8%)	3 (2,4), day 1	Bili	Hospital mortality 30-day mortality
Suzaki 2021	Tokyo, Japan, single center retrospective, observational	n/a	11	6/11 (54.6%)	n/a	n/a	n/a	n/a	Hemolysis and effect on peripheral blood cells in C. perfringens
Talluto 1975	New York, USA, single center, observational	n/a	10****	7/10 (70%)	59.5 (51.75,68)	n/a	n/a	n/a	diagnostic of hemolysis with blood smears
Tanaka 2024	Shiga, Japan, single center, retrospective, observational	Yes	330	28 days: 61/330 (19.1%) 90 days: 95/330 (28.8%) 1 year: 140/330 (42.4%)	Non-schistocyte group (*n* = 289): 74 (63,78) Schistocyte group (*n* = 41): 73 (68,81)	211/330 (63.9%)	8 (6,12) (*n* = 289) 11 (8,14) (*n* = 41) ICU admission SOFA day 1–7 not reported (shown in Figure)	Bili LDH	28-day, 90-day, 1-year mortality, schistocytes as potential markers for massive hemolysis
Wynne 1971	New York, USA single center, retrospective observational	n/a	13	10/13 (76.9%)	48 (37,63)	7/13 (53.8%)	n/a	n/a	patients with neoplasms*****

*CFH* cell-free hemoglobin, *Hp* haptoglobin, *HPX* hemopexin, *Bili* total bilirubin, *LDH* lactate dehydrogenase.

^*^ 9 cases from own medical center, others from literature. **Standard error, ***Values not disclosed. ****Two children excluded. *****One case (16 year) excluded

### Basic study characteristics

A total of 34 studies with an overall of 27,702 patients with sepsis were included and baseline characteristics of included patients are presented in Table 1 [3, 15, 16, 21–51]. An overall mean mortality of 20.1% was observed for all patients with sepsis, ranging between 5.4 and 78.6% [28, 42]. However, mortality was reported differently among studies as ICU-mortality, in-hospital-mortality, 14-day, 28-day-, 90-day-, 180-day, or overall mortality (Table 1). SOFA scores were reported in 17 studies mostly at admission or study enrollment ranging from 2 to 12 (Table 1). Etiology of sepsis was most-commonly associated with respiratory infections, followed by abdominal or surgical infections (Table S1).

### Definition and incidence of hemolysis

**Fig. 2:**
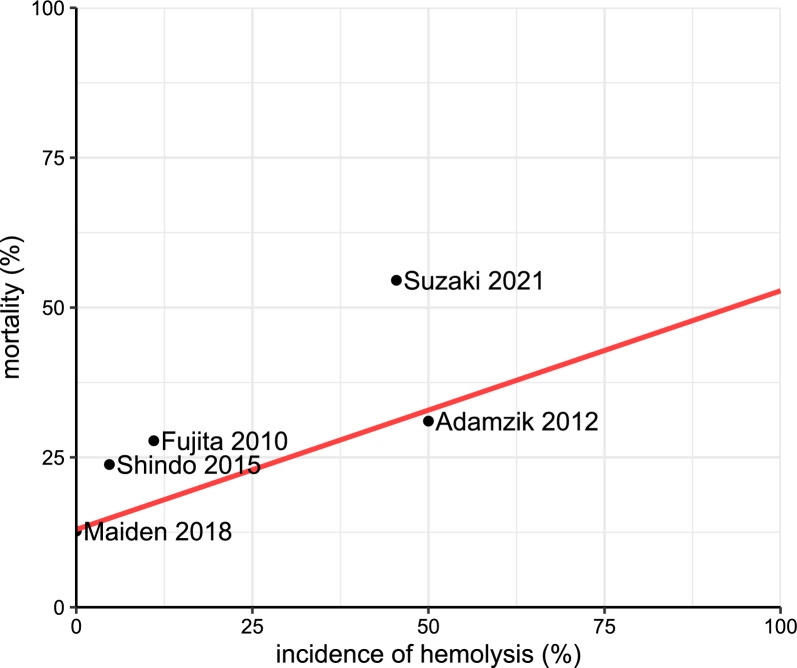
Incidence of self-reported hemolysis and mortality for all studies after 2000. Weighted linear trend line added in red

Definitions of hemolysis ranged from clinical observations, such as jaundice and hemoglobinuria, over microscopic detection of destroyed red blood cells in blood smears, to plasma concentrations of hemolytic markers and were reported in 11 studies (Table 2) [15, 22, 24, 25, 37, 38, 44, 49–52]. The reported incidence of hemolysis ranged from 0 to 50% including only the studies in which the authors defined and identified hemolysis (8 out of 34 studies). All studies published after 2000 are displayed in Fig. 2.

**Table 2:** Reported incidence of hemolysis and hemolytic markers

Study year	Definition of hemolysis	Measurement (time and frequency)	Incidence of hemolysis	Reported averages/values [m—mean ± SD, median (25%, 75% quartiles)]	Impact on mortality	Impact on secondary outcomes
Adamzik 2012	<vs.>median CFH	CFH on day 1 (sepsis/surgery)	80/161 (50%)	mCFH_ELISA_: 0.037 g/l mCFH_Harboe_: 0.068 g/l mCFH_Noe_: 0.085 g/l mCFH_Fairbanks_: 0.059 g/l	Yes	n/a
Boshuizen 2019	n/a	Baseline pre transfusion, 1 h, 24 h	n/a	MedianHp_Bl_: 2.1 (1.0, 3.5) g/l MedianHp_24h_: 2.0 (1.2, 3.5) g/l MedianCFH_Bl_: 6.7 (5.0, 10.5) µmol/l MedianCFH_1h_: 6.9 (4.8, 9.9) µmol/l MedianCFH_24h_: 6.4 (5.1, 10.6) µmol/l MedianFreeHeme_Bl_: 24.3 (19.4, 38.9) µmol/l MedianFreeHeme_1h_: 25.1 (18.9, 36.7) µmol/l MedianFreeHeme_24h_: 24.7 (19.5, 32.4) µmol/l	Unclear	n/a
Caya 1986	Hemoglobinuria and/or elevated CFH (not quantified)	n/a	8/32 (25%)	Not reported	Yes	n/a
Czempik 2023	n/a	Baseline, 4 days, 9 days	n/a	MedianBili: 0.43 (0.28, 0.73) mg/dl	Unclear	n/a
Domizi 2024	CFH	Baseline, 2 h	n/a	medianCFH_Bl_: 1.74 (1.22, 2.94) mg/ml medianCFH_2h_: 2.02 (1.44, 2.95) mg/ml	Unclear	Yes (SOFA with CFH_2h_)
Ekregbesi 2018	Heme, Hp, HPX, HO-1	Once within 12 h of ICU admission	n/a	mHp*: 450 mg/dl mHeme*: 20 µmol/l mHPX*: 11 mg/dl	Yes (heme in regression)	No (APACHE with Hp, Heme or HPX)
Englert 2019	n/a	Once within 24 h of admission	n/a	mBili*: 22 µmol/l MedianHeme survived*: 6 µmol/l MedianHeme deceased*: 7.5 µmol/l	No	No (SOFA (day 1) with Heme/HSA)
Fujita 2010	None	n/a	2/18 (11%)	n/a	No	n/a
Gaini 2007	n/a	Baseline	n/a	mBili: 11.7 ± 6.4 µmol/l	Unclear	n/a
Gaini 2008	n/a	Bili 24-96 h after blood cultures	n/a	mBili: 20.6 ± 26.4 µmol/l	Unclear	n/a
Han 2015	n/a	Baseline, 24 h, 48 h, 72 h	n/a	mHp_Baseline_: 83.0 ± 30.2 mg/dl mHp_24h_: 87.1 ± 47.4 mg/dl mHp_48h_: 93.5 ± 47.0 mg/dl mHp_72h_: 96.9 ± 59.4 mg/dl	Unclear	n/a
Janz 1 2013	n/a**	CFH on enrollment and after 48 h	n/a	MedianCFH_enrollment_: 20 (10, 40)mg/dl MedianCFH_48h_: no acetaminophen: 30 mg/dl vs. acetaminophen: 20 mg/dl, *p* = 0.409	Yes (CFH values + regression)	n/a
Janz 2 2013	n/a	Hp and HPX on enrollment	n/a	MedianCFH: 20 (10, 40) mg/dl MedianHp: 1132 (508, 2890) µg/ml MedianHPX: 591 (383, 925) µg/ml	Yes (CFH, HPX, Hp + regression)	n/a
Janz 2015	n/a	Baseline, once within 3 days	n/a	MedianCFH_baseline_: Treatment: 10 (10, 30) mg/dl Placebo: 20 (10, 32) mg/dl	Unclear	n/a
Kelly 2018	n/a	Baseline, 24 h, 48 h, 72 h	n/a	MedianHp (IQR) in mg/dl and 14-day mortality (survivors vs. non-survivors): baseline: 99.78 (119.43) vs. 99.78 (70.83) 24 h: 99.78 (86.3) vs. 99.78 (81.59) 48 h: 99.78 (148.03) vs. 52.43 (147.61) 72 h: 99.78 (182.52) vs. 67.78 (129.93) MedianHp (IQR) and Hospital-mortality: Baseline: 99.78 (143.03) vs. 99.78 (69.45) 24 h: 99.78 (96.84) vs. 99.78 (73.33) 48 h: 99.78 (201.28) vs. 99.78 (125.47) 72 h: 99.78 (181.43) vs. 99.78 (140.03)	No (not significant)	n/a
Kerchberger 2019	n/a	Study enrollment (Baseline)	Unclear	MedianCFH_HP1-1_: 20 (10, 50) mg/dl MedianCFH_HP2-1_: 20 (10, 30) mg/dl MedianCFH_HP2-2_: 20 (10, 30) mg/dl MedianHp_HP1-1_: 1600 (800, 3880) µg/ml MedianHp_HP1-2_: 1260 (570, 3300) µg/ml MedianHp_HP2-2_: 860 (330, 1940) µg/ml	No	Yes (development of ARDS per CFH quartiles)
Kingston 2020	n/a	Bili at study enrollment	n/a	MedianBili (*n* = 44): 0.4 (0.3, 0.7) mg/dl MedianCFH (*n* = 35): 2.4 (0.7, 5.6) µmol/l	Unclear	n/a
Lan 2022	n/a	Hp at admission	n/a	MedianHp: 159 mg/dl	Yes	Yes (Hp and SOFA)
Larsen 2010	n/a	HPX at septic shock diagnosis + 1 days, 2 days, 3 days, 5 days, 7 days (not reported)	n/a	Median HPX*: 0.75 mg/ml	Yes (28-day mortality and survival time ~ HPX concentration)	Yes (HPX and SOFA in non-survivors)
Leff 1992	Decrease of Hp (not quantified)	Baseline, 6 h, 12 h, 24 h, 48 h	n/a	mBili 2.0 mg/dl mHp*: 223 mg/dl	Unclear	No (development of ARDS and Bili/Hp)
Maiden 2018	Bili (not quantified)	Baseline, 1–6 h, 24 h, 72 h	0/1275 (0%)	Bili unchanged over time, values not reported	Unclear	n/a
Memis 2002	n/a	Baseline, after infusion of methylene blue, 24 h, 48 h	n/a	mBili_Bl_: 1.1 ± 0.22 mg/dl mBili_post MB_: 1.1 ± 0.3 mg/dl mBili_24h_: 1.2 ± 0.2 mg/dl mBili_48h_: 1.1 ± 0.4 mg/dl	Unclear	n/a
Mizuno 2022	n/a	Within 24 h of admission	n/a	MedianHp_All_: 113.2 (50.8, 289) mg/dl MedianHp_low_*** 75 (40.4, 242.1) mg/dl MedianHp_high_*** 123.4 (56.9, 303.7) mg/dl	Yes (in “high HMGB1” Group)	n/a
Nilsson 2020	n/a	Baseline (admission)	Unclear	mBili: 25 ± 41.5 µmol/l	Unclear	n/a
Reah 1997	n/a	Baseline, 24 h, 48 h	n/a	mBili_Bl_ 47 ± 63 µmol/l mBili_24h_ 112 ± 99 µmol/l mBili_48h_ 139 ± 153 µmol/l	Unclear	n/a
Sharma 2019	n/a	Admission, 7 days	Unclear	medianHp_Adm_ survivors vs. non-survivors: 1.6 (0.9, 2.1) mg/ml vs. 1.3 (0.9, 1.7) mg/ml medianHp_d7_ survivors vs. non-survivors: 1.4 (0.8, 1.7) mg/ml vs. 1.5 (1.2, 1.6) mg/ml	No	n/a
Shindo 2015	Bili (total and direct), LDH (not quantified)	n/a	1/21 (4.7%)	Bili_total_****: 10.34 mg/dl Bili_direct_: 5.84 mg/dl LDH: 6138 U/l	No	n/a
Spies 1994	n/a	Baseline	Unclear	mBili: 80 ± 79 µmol/l	Unclear	n/a
Staudinger 1996	n/a	Baseline, 24 h	Unclear	mHp_Bl_ 296.5 ± 161 mg/dl mHp_24h_ 288 ± 162 mg/dl	Unclear	n/a
Sun 2024	n/a	Bili at admission	n/a	medianBili: 0.73 (0.50, 1.15) µmol/l	Unclear	n/a
Suzaki 2021	Serum appeared exceptionally bright red with marked hemoglobinemia	Unclear	5/11 (45.45%)	n/a	Yes	n/a
Talluto 1975	Visual examination of Hct, spherocytes, microcytes, schistocytes, ghost RBCs	Admission	1/10 (10%)	n/a	No	n/a
Tanaka 2024	Bili, LDH (not quantified)	Bili, LDH at admission and in the first 7 days	n/a	Non-schistocyte group vs. schistocyte group: MedianBili_Admission_: 0.9 (0.5, 1.3) mg/dl vs. 0.7 (0.5, 1.2) mg/dl MedianLDH_Admission_: 249 (190, 378) U/l vs. 282.5 (215, 417.5) U/l MedianBili_d1-7_:* 1 mg/dl vs. 1.5 mg/dl, *p* < 0.05 MedianLDH_d1-7_*: 250 U/l	Unclear	n/a
Wynne 1971	Jaundice, Hct ↓ & hemoglobinuria	n/a	2/13 (15.4%)	Not reported	Unclear	n/a

*CFH* cell-free hemoglobin, *Hp* haptoglobin, *HPX* hemopexin, *Bili* total Bilirubin, *LDH* lactate dehydrogenase

^*^ Values approximated from figure

^**^ Nine hemolyzed samples (enrollment) were excluded, definition for hemolysis or CFH threshold of excluded samples were not reported. No clinical data on excluded patients provided

^***^ Low = under median, high = over median

^****^ Reported value from one patient (with hemolysis)

### Levels of hemolytic markers

**Fig. 3:**
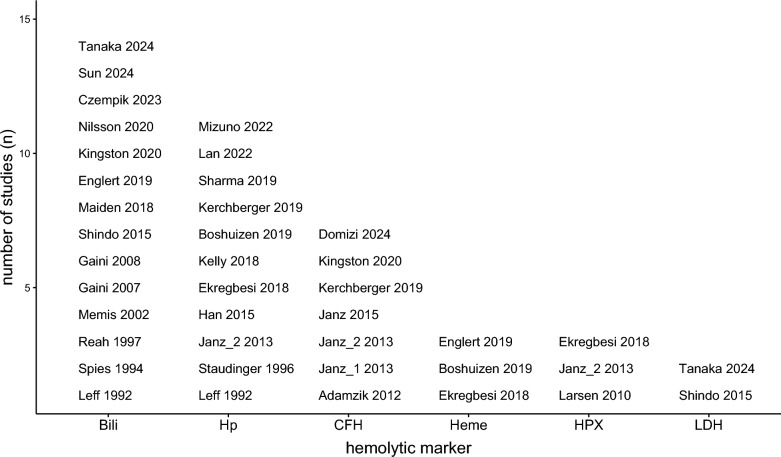
Reported hemolytic markers

In 28 studies, plasma concentrations of hemolytic markers were reported with the most common marker being bilirubin (14 studies), followed by Hp (11 studies), CFH (7 studies), Hx (3 studies), free heme (3 studies) and lactate dehydrogenase (LDH) (2 studies) (Fig. 3). Most studies only reported initial values at ICU admission, at sepsis diagnosis, or at study enrollment. Repeated values over the course of sepsis were only mentioned in 14 studies (Table 2).

### Association of hemolysis and mortality

An association between hemolysis or hemolytic markers and increased mortality was reported in nine studies. Significant associations between elevated CFH levels and mortality were observed by the groups of Caya, Adamzik and Janz [15, 16, 22, 31]. For the hemolysis marker Hp, 11 studies reported data in connection with mortality but only the groups of Janz, Mizuno, and Lan observed a significant association of decreased Hp with increased mortality in patients with sepsis (Table 2, Table S2) [21, 25, 30, 31, 33, 34, 36, 37, 40, 43, 46]. Janz and colleagues demonstrated that median Hp plasma concentrations on study enrollment were significantly lower in non-survivors compared to survivors which was robust in a univariate logistic regression and a multivariate regression analysis for Hp, CFH and mortality [31]. For patients without elevated CFH however, median Hp plasma concentration did not differ between non-survivors and survivors [31]. In a cohort of septic shock patients, Lan and colleagues categorized the patients into three groups according to the tertiles of Hp values also finding higher Hp plasma concentrations associated with improved 28-day, 90-day, ICU, and hospital mortality [36]. A multivariable Cox proportional hazard model underlined the lower risk of 28-day mortality associated with increased Hp plasma concentrations [36]. Mizuno demonstrated that patients with sepsis and Hp plasma concentrations below 109 mg/dl had a poorer long-term prognosis measured by 180-day mortality compared to patients with sepsis and higher Hp plasma concentrations [40].

In the study of Ekregbesi et al., Hx plasma concentrations within 12 h of ICU admission did not show an association with mortality [25]. In contrast, Larsen and colleagues reported that reduced mean Hx plasma concentrations were associated with increased 28-day mortality in patients with sepsis [3]. Moreover, survival time appeared proportional to Hx plasma concentrations. The significant association of reduced Hx levels with increased mortality in septic patients was supported by the data presented by Janz and colleagues [31]. Like for Hp, univariate and multivariate regression analysis for Hp, CFH and mortality suggested an association of lower Hx plasma concentrations with an increased mortality in septic patients. However, no association with regard to mortality was detected for different Hx plasma concentrations when septic patients did not have elevated plasma concentrations of CFH [31].

Due to the technical difficulty of the measurement in clinical routine, only three studies reported data on free heme [21, 25, 26]. However, only Ekregbesi and colleagues reported outcome data related to mortality with a hazard ratio of a 4.04 for an increased mortality for patients whose free heme concentration, measured within 12 h of ICU admission, was above the median heme concentration of all patients (median heme concentration reported with 21.1 μM) [25].

For the frequently measured serum bilirubin, no association with mortality was reported in all 14 studies except for the study of Shindo and colleagues that reported a higher mortality with high bilirubin concentrations (above 10.34 mg/dl) [44]. However, this was found in only one out of 21 subjects. Shindo et al. were also the only ones reporting data for LDH, also in one patient out of 21 and claiming that elevated LDH concentrations were a sign of hemolysis and associated with increased mortality [44]. In contrast, the largest study defining hemolysis by increased plasma concentrations of bilirubin by Maiden and colleagues encompassing 1275 patients and retrospective data could not detect hemolysis or a positive correlation between increased plasma concentrations of bilirubin and mortality [38].

### Association of hemolysis with organ failure

Domizi and colleagues found a weak correlation between CFH and the SOFA score in 50 patients with a calculated Spearman’s correlation coefficient of 0.306, *p* = 0.032 at 2 h after acetaminophen administration [24]. A significant inverse correlation between Hx levels (on day 3 and 5 after sepsis diagnosis) and SOFA scores in non-survivors was observed by Larsen and colleagues (Spearman´s correlation coefficient of − 0.58, *p* = 0.048 on day 3 and − 0.93, *p* = 0.002 on day 5 for non-survivors) [3]. Lan et al. showed that increases in SOFA score were associated with a gradual decrease in plasma concentrations of Hp measured on ICU admission in patients with sepsis [36]. Leff and colleagues reported that higher serum bilirubin concentrations in patients with sepsis were associated with increased likelihood for development of an ARDS [37]. Similarly, Kerchberger et al. described a successively increased rate of development of ARDS with increased plasma concentrations of CFH in patients with sepsis [34]. They calculated an odds ratio of 1.12 per 50 mg/dl CFH for increased chances of ARDS development [34].

### Potential confounders of hemolysis

The number and age of transfused PRBCs was taken into consideration as potential confounders of hemolysis by six studies (Table 3) [15, 16, 21, 31, 41, 50]. Therapy with RRT was considered in 10 studies (Table 3) [15, 16, 23, 31–33, 36, 41, 47, 50]. Several studies reported data on the frequency of treatment with dialysis on ICU admission (Table 3). However, duration of hemodialysis was not reported (Table 3). In contrast, therapy with an extracorporeal life support (ECLS) was considered in none of all identified studies. There were 12 studies reporting data on specific pathogens associated with the sepsis (Table 3). Three additional studies reported specifics on the gram staining of the pathogens while in four of the included studies, alpha-hemolytic streptococcus species were identified pathogens (Table 3). Clostridium species were identified in eight studies of which three studies reported data on clostridium perfringens (Table 3).

**Table 3:** PRBCs, RRT, ECLS and pathogens

Study year	No. of PRBCs	RRT, *n* (%), time	ECLS (%)	Infection type/pathogen	Corrected for in mortality or hemolysis analysis
Adamzik 2012	PRBCs within 3 days	91 (56.5%), n/a	n/a	Gram positive: 40.4% Gram negative: 35.0% Fungal: 15.5% Culture negative: 9.1%	No (mortality) “negative correlation” with CFH
Boshuizen 2019	1Unit, in 13 (6–22) days	n/a	n/a	n/a	n/a (mortality) ΔHp(%)_PRBC_: − 2.7 (− 7.1–8.1) ΔCFH(%)_PRBC_: 1.7 (− 5.9–14.4) ΔFreeHeme(%)_PRBC_: 3.3 (− 8.2–7.8)
Caya 1986	n/a	n/a	n/a	*C.* spp. 100%	n/a
Czempik 2023	n/a	7 (20.0%), n/a	n/a	n/a	n/a
Domizi 2024	n/a	n/a	n/a	Multiple drug resistant 20% Polymicrobial 16% Negative microbiology 14%	n/a
Ekregbesi 2018	n/a	n/a	n/a	n/a	n/a
Englert 2019	n/a	n/a	n/a	Non-identified 33.3% *S. aureus* 22.2% *E. coli* 22.2% *E. faecalis* 16.7% *E. faecium* 11.1% *P. aeruginosa* 5.6% *Enterococcus* 5.6% *C. diff*. 5.6% *	n/a
Fujita 2010	n/a	n/a	n/a	*C*. spp 100%	No
Gaini 2007	n/a	n/a	n/a	Gram+ 29.8% Gram− 23.0% Viral 6.8%	n/a
Gaini 2008	n/a	n/a	n/a	Gram+ 51.2% gram− 45.3% Polymicrobial 3.5%	n/a
Han 2015	n/a	n/a	n/a	Bacterial 100% (*C. difficile* 3.2%)	n/a
Janz 1 2013	91 (23.3%), No. unknown	14 (3.6%), enrollment	n/a	n/a	Mortality: RRT OR 0.215 [95% CI 0.025–1.838], *p* = 0.16, CFH_adj_** (per 10 mg/dl) OR 1.078 [1.012–1.149], *p* = 0.02
Janz 2 2013	90 (23.3%), No. unknown	14 (3.6%), enrollment	n/a	n/a	No
Janz 2015	n/a	4 (10.0%), n/a	n/a	n/a	No
Kelly 2018	n/a	8 (5.8%), n/a	n/a	Bacteremia 64.0%	No
Kerchberger 2019	n/a	n/a, n/a	n/a	n/a	n/a
Kingston 2020	n/a	n/a	n/a	n/a	n/a
Lan 2022	n/a, n/a	45 (9.0%), n/a	n/a	n/a	No
Larsen 2010	n/a, n/a	n/a	n/a	Positive microbial cultures 65%	No
Leff 1992	n/a	n/a	n/a	n/a	n/a
Maiden 2018	None	n/a	n/a	n/a	n/a
Memis 2002	n/a	n/a	n/a	*P. aeruginosa* 36.7% *S. aureus* 20.0% *E. coli* 16.7% *K. pneumoniae* 10.0% *S. pneumoniae* 10.0% *E. faecalis* 10.0%*	n/a
Mizuno 2022	n/a	n/a	n/a	n/a	n/a
Nilsson 2020	1–2 units vs. 0 in 5 days	69 (14.6%), n/a	n/a	n/a	Yes (mortality), n/a (hemolysis)
Reah 1997	n/a	n/a	n/a	n/a	n/a
Sharma 2019	n/a	n/a	n/a	n/a	n/a
Shindo 2015	n/a	n/a	n/a	*C. perfringens* 100%	n/a
Spies 1994	n/a	n/a	n/a	n/a	n/a
Staudinger 1996	n/a	None (exclusion criterion)	n/a	Unspecified 41.7% *Candida* 16.6% *S. aureus* 12.5% *S. pneumoniae* 8.3% *Meningococci* 8.3% *Klebsiella pneumoniae* 4.2% *E. coli* 4.2% *P. aeruginosa* 4.2% CMV 4.2% *	n/a
Sun 2024	n/a	1199 (5.3%), admission	n/a	n/a	Yes (mortality), n/a (hemolysis)
Suzaki 2021	None	n/a	n/a	*C. perfringens* 100%	n/a
Talluto 1975	n/a	n/a	n/a	*E. coli* 30.0% *N. meningitides* 20.0% *Bacteroides* 20.0% *D. pneumoniae* 10.0% *C. perfringens* 10.0% *P. mirabilis* 10.0%	n/a
Tanaka 2024	0.5 units/day vs. 0.9 units/day	244 (73.9%), admission	n/a	n/a	No
Wynne 1971	n/a	n/a	n/a	*C.* spp. 100%	n/a

^*^ Infections with more than one pathogen reported

^**^ Adjusted for RRT, age, APACHE Score, chronic liver disease

^***^ Exclusion criterion

## Discussion

In this Scoping Review, 34 studies were assessed with 24% reporting incidences of hemolysis in patients with sepsis. There were 18 studies reporting data on specific laboratory markers of hemolysis (CFH, Hp, Hx, free Heme) and an association between hemolysis or hemolytic markers and increased mortality was reported in nine studies.

The definition of hemolysis is heterogeneous and encompasses clinical findings like red coloration of urine or jaundice which was mainly reported in early studies, configuration of blood smears, and varying concentrations of different markers with various specificity for hemolysis such as CFH, Hp, Hx, free heme, and serum bilirubin. Based on the heterogeneity of monitoring strategies, the incidence of self-reported detected hemolysis in patients with sepsis showed a very high variation between the retrieved articles ranging from 0–50%.

As a summary from the synoptic evaluation of the reported hemolytic markers, higher plasma concentrations of CFH, free heme, and serum bilirubin and in contrast lower plasma concentrations of Hp and Hx appear associated with increased mortality in patients with sepsis. Only few of the identified studies reported data on further organ failure or impairment associated with sepsis and hemolysis such as associations with increases in SOFA-Score or development of ARDS [34, 36, 37].

Considering the above-mentioned associations of hemolysis with mortality in patients with sepsis, several hemolysis markers might be useful for evaluation of disease severity or even for prognostication in patients with sepsis. However, in most studies, hemolysis markers were only measured once at study enrollment or at ICU admission, seldom multiple times and never beyond the first week of sepsis treatment. Based on the observational character of most studies, the degree of hemolysis was mainly observed and considered as a marker of disease severity. From data in patients with ARDS and treatment with veno-venous ECMO, it is known that decreases of plasma concentrations of Hp within the first week of treatment with ECMO were associated with an increased 28-day mortality [53]. In the light of hemolysis as an ongoing process during the course of disease and the potential effects and implications of therapeutic interventions on the degree of hemolysis, markers of hemolysis might also contribute to the continuous evaluation of disease severity over therapy time. This is further underlined by the noticed association with hemolysis and increases in the SOFA-Score [3, 24, 36].

Not measuring indicators of hemolysis during the course of the disease might lead to undetected cases of hemolysis occurring at later timepoints that might be associated or caused by disease progression or various therapeutic interventions knowingly associated with intravascular hemolysis such as transfusions of PRBCs, RRT or therapy with ECMO [17–19, 54]. This may result in an underestimation and underreporting of hemolysis and hemolytic events. In their treatment guidelines for patients receiving therapy with ECMO, the Extracorporeal Life Support Organization (ELSO) defines “moderate hemolysis” as a CFH value exceeding 50 mg/dl for two consecutive days [55]. Relevant increases in CFH can occur during the entire course of therapy with ECMO. However, even CFH plasma concentrations greater then 11 mg/dl were lately shown to be associated with increased 28-day mortality in patients with ARDS and therapy with ECMO [18].

While most studies only reported values of hemolysis markers at sepsis diagnosis, ICU admission, or study enrollment, only few studies reported additional values over the course of sepsis with even fewer studies reporting data for consecutive days on the ICU. During the dynamic course of critical illness on the ICU, also the dynamic changes in monitored biochemical markers and physiological parameters might allow higher quality evaluation and prognostication of disease states. Again, knowing from patients treated with ECMO for severe ARDS, dynamic decreases of hemolysis marker Hp are of significant prognostic value [53]. So far, no study provided daily sequential and consecutive measurements of hemolysis markers during the entire course of ICU treatment.

Furthermore, plasma concentrations of CFH and its breakdown products free heme and bilirubin should always be evaluated with respect to the plasma concentrations of the endogenous CFH-opponent Hp and the heme-binding protein Hx. In patients with ARDS and treatment with veno-venous ECMO, high endogenous plasma concentrations of Hp could compensate for the negative effects of disease- and therapy-associated high plasma concentrations of CFH [17]. This connection so far has not been addressed in the studies on patients with sepsis.

Based on the data retrieved for this review, serum Hp levels below 109 mg/dl were associated with a higher mortality after 90 or more days after ICU admission in patients with sepsis while Hp levels above 215 mg/dl showed a significant survival benefit [36, 40]. For CFH, the study of Janz and colleagues demonstrated that CFH values above 20 mg/dl were associated with an increased hospital mortality compared to CFH values below 10 mg/dl [16]. However, the other studies addressing CFH as surrogate parameter for hemolysis did not define any cut-off values for CFH and only stated that higher values were associated with poorer outcomes. Similarly, the studies focusing on further hemolysis parameters such as Hx, heme, bilirubin, or LDH did not determine cut-off values for the reported markers of hemolysis that are associated with adverse outcomes in patients with sepsis.

### Strengths and limitations

This scoping review includes 34 studies with 27,702 patients with sepsis that addressed hemolysis in single or multicenter, retrospective or prospective, and observational or interventional trails. However, due to the high heterogeneity of the included studies in design and methodology, the different definitions and quantifications of hemolysis and the heterogeneous and inconsistently chosen markers of hemolysis, a meta-analysis could not be performed. Most of the included studies encompassed single-point measurements of hemolytic markers or associated clinical read-outs such as the SOFA-Score which was calculated heterogeneously in the different studies on admission, on study inclusion or at even later time points. Given the dynamic course of sepsis, serial measurements of those markers might demonstrate a more comprehensive picture of the clinical relevance of hemolysis in critically ill patients. Furthermore, the biomarkers, that were chosen in many of the included studies to assess the degree of hemolysis, are often affected by multiple cofounders. Bilirubin, the most frequently chosen but indirect marker for hemolysis, is also an indicator of liver dysfunction and affected by liver organ damage which frequently occurs in sepsis [56]. Some of the studies corrected for liver dysfunction also measuring further biomarkers such as aspartate transaminases (AST), alanine transaminases (ALT), direct and indirect bilirubin, and glutamate-pyruvate transaminases [23, 32, 39, 44, 47, 50]. Hp as a direct marker of hemolysis is also frequently known as an acute phase protein whose synthesis might interfere with proinflammatory settings present in patients with sepsis [57]. Furthermore, Hp occurs in different genetic variants with different physiological properties [58, 59]. The clinical relevance of these different Hp genetic variants is currently under investigation. However, preclinical research strongly suggests a different potency of the genetic variants regarding the binding and degradation of the CFH-Hp-complexes. For example, Hp1-1 forms the strongest bond to CFH, but both, Hp 2–1 and Hp 2–2 can bind a higher number of alpha–beta dimers of CFH. The complex of CFH with the genetic variant Hp 2–2 has the highest affinity to the CD163 receptor which initiates internalization of the complex into the macrophages with consecutive degradation [60]. In patients with sepsis, the different Hp genotypes are associated with different susceptibilities for development of ARDS [34]. The strongest direct biomarker for hemolysis, CFH, was only measured in seven of the 34 included studies. Interferences with the technical ways of measuring CFH in routine laboratory diagnostics were addressed by Adamzik and colleagues [15]. Furthermore, and besides the above-mentioned interferences of CFH with serum Hp, CFH undergoes a hepatic and renal elimination with direct filtration of CFH through the renal glomerulus [13]. Therefore, plasma clearance and concentrations are directly associated with renal function that is often compromised in patients with sepsis and septic shock [61]. Finally, the included studies generally did not adjust for factors such as comorbidities or intensive care medical treatments and medications which all can interfere with plasma concentrations of biomarkers for hemolysis.

Given the high heterogeneity of both, specific markers of hemolysis (CFH, Hp, Hx and free Heme) and markers potentially associated with hemolysis but lacking specificity (Bili, LDH), also the definition of hemolysis varied widely across the included studies limiting comparability and interpretability of the results. Additionally, the reported CFH values rely on either averages of the investigated markers comparing survivors or non-survivors or of medians of the investigated cohort but were not calculated for prognostic or future use in a different cohort. Future studies addressing hemolysis in critically ill patients should focus on the specific markers of hemolysis and possible cut-off values might have to be defined in a consensus conference or calculated in large data cohorts.

## Conclusions

Although significant associations between hemolysis and outcome in patients with sepsis were observed, hemolytic markers are not yet regularly monitored in the clinical routine on the ICU. Hemolytic markers can provide information about disease severity and outcome on ICU admission and during the course of the disease. Both, the direct markers of hemolysis such as CFH, free heme, Hp, and Hx and the indirect markers like bilirubin and LDH are all and differently affected by various comorbidities, complications and therapeutic interventions. Future work should focus on identification of reliable markers of hemolysis with a potential for easy and timely measurements ideally at the patients’ bedside. With an additional definition of monitoring standards, the potential of hemolysis monitoring for prognostication and therapeutic approaches will emerge.

## Take home message

In clinical routine, hemolysis is often overlooked, as markers of hemolysis are often not frequently measured. In patients with sepsis, hemolytic markers can provide information about disease severity and outcome.

## Supplementary Information

Additional file 1.

## Data Availability

Data are available from the corresponding author on reasonable request.

## Abbreviations

ARDS: Acute respiratory distress syndrome
CFH: Cell-free hemoglobin
Hp: Haptoglobin
HPX: Hemopexin
Bili: Total bilirubin
LDH: Lactate dehydrogenase
NO: Nitric oxide
SOFA: Sequential Organ Failure Assessment
APACHE: Acute Physiology and Chronic Health Evaluation
RRT: Renal replacement therapy
PRBC: Packed red blood cells

## References

[1] Rudd KE, Johnson SC, Agesa KM et al (2020) Global, regional, and national sepsis incidence and mortality, 1990–2017: analysis for the Global Burden of Disease study. Lancet 395:200–211 31954465 10.1016/S0140-6736(19)32989-7 PMC6970225

[2] Sakr Y, Jaschinski U, Wittebole X et al (2018) Sepsis in intensive care unit patients: worldwide data from the Intensive Care over Nations Audit. Open Forum Infect Dis 5:ofy313 30555852 10.1093/ofid/ofy313 PMC6289022

[3] Larsen R, Gozzelino R, Jeney V et al (2010) A central role for free heme in the pathogenesis of severe sepsis. Sci Transl Med 2:51ra71 10.1126/scitranslmed.3001118 20881280

[4] Amaral A, Opal SM, Vincent J-L (2004) Coagulation in sepsis. Intensive Care Med 30:1032–1040 15148567 10.1007/s00134-004-2291-8

[5] Effenberger-Neidnicht K, Hartmann M (2018) Mechanisms of hemolysis during sepsis. Inflammation 41:1569–1581 29956069 10.1007/s10753-018-0810-y

[6] Balla J, Vercellotti GM, Jeney V et al (2007) Heme, heme oxygenase, and ferritin: how the vascular endothelium survives (and dies) in an iron-rich environment. Antioxid Redox Signal 9:2119–2138 17767398 10.1089/ars.2007.1787

[7] Donadee C, Raat NJH, Kanias T et al (2011) Nitric oxide scavenging by red blood cell microparticles and cell-free hemoglobin as a mechanism for the red cell storage lesion. Circulation 124:465–476 21747051 10.1161/CIRCULATIONAHA.110.008698 PMC3891836

[8] Villagra J, Shiva S, Hunter LA, Machado RF, Gladwin MT, Kato GJ (2007) Platelet activation in patients with sickle disease, hemolysis-associated pulmonary hypertension, and nitric oxide scavenging by cell-free hemoglobin. Blood 110:2166–2172 17536019 10.1182/blood-2006-12-061697 PMC1976348

[9] Jeney V, Balla J, Yachie A et al (2002) Pro-oxidant and cytotoxic effects of circulating heme. Blood 100:879–887 12130498 10.1182/blood.v100.3.879

[10] Lin T, Kwak YH, Sammy F et al (2010) Synergistic inflammation is induced by blood degradation products with microbial Toll-like receptor agonists and is blocked by hemopexin. J Infect Dis 202:624–632 20617898 10.1086/654929 PMC2932749

[11] Hod EA, Zhang N, Sokol SA et al (2010) Transfusion of red blood cells after prolonged storage produces harmful effects that are mediated by iron and inflammation. Blood 115:4284–4292 20299509 10.1182/blood-2009-10-245001 PMC2879099

[12] Thomsen JH, Etzerodt A, Svendsen P, Moestrup SK (2013) The Haptoglobin-CD163-heme oxygenase-1 pathway for hemoglobin scavenging. Oxid Med Cell Longev 2013:1–11 10.1155/2013/523652 PMC3678498 23781295

[13] Vallelian F, Buehler PW, Schaer DJ (2022) Hemolysis, free hemoglobin toxicity, and scavenger protein therapeutics. Blood 140:1837–1844 35660854 10.1182/blood.2022015596 PMC10653008

[14] Rother RP, Bell L, Hillmen P, Gladwin MT (2005) The clinical sequelae of intravascular hemolysis and extracellular plasma hemoglobin: a novel mechanism of human disease. JAMA 293:1653 15811985 10.1001/jama.293.13.1653

[15] Adamzik M, Hamburger T, Petrat F, Peters J, De Groot H, Hartmann M (2012) Free hemoglobin concentration in severe sepsis: methods of measurement and prediction of outcome. Crit Care 16:R125 22800762 10.1186/cc11425 PMC3580706

[16] Janz DR, Bastarache JA, Peterson JF et al (2013) Association between cell-free hemoglobin, acetaminophen, and mortality in patients with sepsis: an observational study*. Crit Care Med 41:784–790 23314583 10.1097/CCM.0b013e3182741a54 PMC3578977

[17] Graw JA, Hildebrandt P, Krannich A et al (2022) The role of cell-free hemoglobin and haptoglobin in acute kidney injury in critically ill adults with ARDS and therapy with VV ECMO. Crit Care 26:50 35193645 10.1186/s13054-022-03894-5 PMC8864920

[18] Bünger V, Hunsicker O, Krannich A et al (2023) Potential of cell-free hemoglobin and haptoglobin as prognostic markers in patients with ARDS and treatment with veno-venous ECMO. J Intensive Care 11:15 37081577 10.1186/s40560-023-00664-5 PMC10116665

[19] Materne LA, Hunsicker O, Menk M, Graw JA (2021) Hemolysis in patients with extracorporeal membrane oxygenation therapy for severe acute respiratory distress syndrome - a systematic review of the literature. Int J Med Sci 18:1730–1738 33746589 10.7150/ijms.50217 PMC7976579

[20] Ouzzani M, Hammady H, Fedorowicz Z, Elmagarmid A (2016) Rayyan—a web and mobile app for systematic reviews. Syst Rev 5:210 27919275 10.1186/s13643-016-0384-4 PMC5139140

[21] Boshuizen M, Van Hezel ME, Van Manen L et al (2019) The effect of red blood cell transfusion on iron metabolism in critically ill patients. Transfusion 59:1196–1201 30597563 10.1111/trf.15127

[22] Caya JG, Farmer SG, Ritch PS et al (1986) Clostridial septicemia complicating the course of leukemia. Cancer 57:2045–2048 3456820 10.1002/1097-0142(19860515)57:10<2045::aid-cncr2820571028>3.0.co;2-o

[23] Czempik PF, Wiórek A (2023) Iron deficiency in sepsis patients managed with divided doses of iron dextran: a prospective cohort study. Sci Rep 13:5264 37002279 10.1038/s41598-023-32002-y PMC10066317

[24] Domizi R, Damiani E, Carsetti A et al (2024) Potential of acetaminophen on the sublingual microcirculation and peripheral tissue perfusion of febrile septic patients: prospective observational study. Ann Intensive Care 14:23 38340203 10.1186/s13613-024-01251-z PMC10858855

[25] Ekregbesi P, Shankar-Hari M, Bottomley C, Riley EM, Mooney JP (2018) Relationship between anaemia, haemolysis, inflammation and haem oxygenase-1 at admission with sepsis: a pilot study. Sci Rep 8:11198 30046137 10.1038/s41598-018-29558-5 PMC6060141

[26] Englert FA, Seidel RA, Galler K et al (2019) Labile heme impairs hepatic microcirculation and promotes hepatic injury. Arch Biochem Biophys 672:108075 31412260 10.1016/j.abb.2019.108075

[27] Fujita H, Nishimura S, Kurosawa S, Akiya I, Nakamura-Uchiyama F, Ohnishi K (2010) Clinical and epidemiological features of *Clostridium perfringens* bacteremia: a review of 18 cases over 8 year-period in a tertiary care center in metropolitan Tokyo area in Japan. Intern Med 49:2433–2437 21088344 10.2169/internalmedicine.49.4041

[28] Gaïni S, Pedersen S, Koldkjær O, Pedersen C, Møller H (2007) High mobility group box-1 protein in patients with suspected community-acquired infections and sepsis: a prospective study. Crit Care 11:R32 17346334 10.1186/cc5715 PMC2206448

[29] Gaïni S, Pedersen SS, Koldkjær OG, Pedersen C, Moestrup SK, Møller HJ (2008) New immunological serum markers in bacteraemia: anti-inflammatory soluble CD163, but not proinflammatory high mobility group-box 1 protein, is related to prognosis. Clin Exp Immunol 151:423–431 18190604 10.1111/j.1365-2249.2007.03586.x PMC2276958

[30] Han JH, Nachamkin I, Coffin SE et al (2015) Use of a combination biomarker algorithm to identify medical intensive care unit patients with suspected sepsis at very low likelihood of bacterial infection. Antimicrob Agents Chemother 59:6494–6500 26239984 10.1128/AAC.00958-15 PMC4576082

[31] Janz DR, Bastarache JA, Sills G et al (2013) Association between haptoglobin, hemopexin and mortality in adults with sepsis. Crit Care 17:R272 24225252 10.1186/cc13108 PMC4056258

[32] Janz DR, Bastarache JA, Rice TW et al (2015) Randomized, placebo-controlled trial of acetaminophen for the reduction of oxidative injury in severe sepsis: the Acetaminophen for the Reduction of Oxidative Injury in Severe Sepsis Trial*. Crit Care Med 43:534–541 25474535 10.1097/CCM.0000000000000718 PMC4336619

[33] Kelly BJ, Lautenbach E, Nachamkin I et al (2018) Combined biomarkers predict acute mortality among critically ill patients with suspected sepsis*. Crit Care Med 46:1106–1113 29912095 10.1097/CCM.0000000000003137 PMC6010038

[34] Kerchberger VE, Bastarache JA, Shaver CM et al (2019) Haptoglobin-2 variant increases susceptibility to acute respiratory distress syndrome during sepsis. JCI Insight 4:e131206 31573976 10.1172/jci.insight.131206 PMC6948757

[35] Kingston HWF, Ghose A, Rungpradubvong V et al (2019) Cell-free hemoglobin is associated with increased vascular resistance and reduced peripheral perfusion in severe malaria. J Infect Dis 221:jiz359 10.1093/infdis/jiz359 31693729

[36] Lan P, Yu P, Ni J, Zhou J (2022) Higher serum haptoglobin levels were associated with improved outcomes of patients with septic shock. Crit Care 26:131 35578264 10.1186/s13054-022-04007-y PMC9112476

[37] Leff JA, Parsons PE, Day CE et al (1992) Increased serum catalase activity in septic patients with the adult respiratory distress syndrome. Am Rev Respir Dis 146:985–989 1416429 10.1164/ajrccm/146.4.985

[38] Maiden MJ, Finnis ME, Peake S et al (2018) Haemoglobin concentration and volume of intravenous fluids in septic shock in the ARISE trial. Crit Care 22:118 29724246 10.1186/s13054-018-2029-6 PMC5934793

[39] Memis D, Karamanlioglu B, Yuksel M, Gemlik I, Pamukcu Z (2002) The influence of methylene blue infusion on cytokine levels during severe sepsis. Anaesth Intensive Care 30:755–762 12500513 10.1177/0310057X0203000606

[40] Mizuno T, Eguchi Y, Tsujita Y, Imashuku Y, Tabata T, Kitagawa H (2022) Mortality at 180-days is affected by serum haptoglobin levels in septic patients with high magnitude serum high mobility group box-1 levels. Acute Med Surg 9:e726 35127103 10.1002/ams2.726 PMC8805693

[41] Nilsson CU, Bentzer P, Andersson LE, Björkman SA, Hanssson FP, Kander T (2020) Mortality and morbidity of low-grade red blood cell transfusions in septic patients: a propensity score-matched observational study of a liberal transfusion strategy. Ann Intensive Care 10:111 32770427 10.1186/s13613-020-00727-y PMC7415067

[42] Reah G, Bodenham AR, Mallick A, Daily EK, Przybelski RJ (1997) Initial evaluation of diaspirin cross-linked hemoglobin (DCLHb[trademark symbol]) as a vasopressor in critically ill patients. Crit Care Med 25:1480–1488 9295821 10.1097/00003246-199709000-00014

[43] Sharma NK, Ferreira BL, Tashima AK et al (2019) Lipid metabolism impairment in patients with sepsis secondary to hospital acquired pneumonia, a proteomic analysis. Clin Proteomics 16:29 31341447 10.1186/s12014-019-9252-2 PMC6631513

[44] Shindo Y, Dobashi Y, Sakai T, Monma C, Miyatani H, Yoshida Y (2015) Epidemiological and pathobiological profiles of *Clostridium**perfringens* infections: review of consecutive series of 33 cases over a 13-year period. Int J Clin Exp Pathol 8:569 25755747 PMC4348875

[45] Spies CD, Reinhart K, Witt I et al (1994) Influence of N-acetylcysteine on indirect indicators of tissue oxygenation in septic shock patients: results from a prospective, randomized, double-blind study. Crit Care Med 22:1738–1746 7956276

[46] Staudinger T, Presterl E, Graninger W et al (1996) Influence of pentoxifylline on cytokine levels and inflammatory parameters in septic shock. Intensive Care Med 22:888–893 8905422 10.1007/BF02044112

[47] Sun S, Liu H, Liang Q, Yang Y, Cao X, Zheng B (2024) Association between acetaminophen administration and clinical outcomes in patients with sepsis admitted to the ICU: a retrospective cohort study. Front Med 11:1346855 10.3389/fmed.2024.1346855 PMC10864567 38357644

[48] Suzaki A, Komine-Aizawa S, Nishiyama H, Hayakawa S (2022) Massive intravascular hemolysis is an important factor in *Clostridium perfringens*-induced bacteremia. Intern Emerg Med 17:1959–1967 35962901 10.1007/s11739-022-03036-3

[49] Talluto MR (1975) Hematological findings in acute infections and septicemias. Am J Med Technol 41:377–386 242218

[50] Tanaka T, Fujino K, Tsujita Y et al (2024) The impact of schistocyte detection on mortality and organ failure in patients with sepsis. Shock 62:539–546 39158562 10.1097/SHK.0000000000002440

[51] Wynne JW, Armstrong D (1972) Clostridial septicemia. Cancer 29:215–221 4550205 10.1002/1097-0142(197201)29:1<215::aid-cncr2820290132>3.0.co;2-c

[52] Suzaki A, Ohtani K, Komine-Aizawa S, Matsumoto A, Kamiya S, Hayakawa S (2021) Pathogenic characterization of *Clostridium perfringens* strains isolated from patients with massive intravascular hemolysis. Front Microbiol 12:713509 34385995 10.3389/fmicb.2021.713509 PMC8353389

[53] Bünger V, Hunsicker O, Krannich A et al (2024) Haptoglobin depletion during the first seven days of veno-venous ECMO therapy is associated with increased mortality and adverse outcomes in patients with ARDS. Shock 61:828–835 38661177 10.1097/SHK.0000000000002352

[54] Hod EA, Brittenham GM, Billote GB et al (2011) Transfusion of human volunteers with older, stored red blood cells produces extravascular hemolysis and circulating non–transferrin-bound iron. Blood 118:6675–6682 22021369 10.1182/blood-2011-08-371849 PMC3242722

[55] Extracorporeal Life Support Organization (2022) ELSO registry data definitions. Extracorporeal Life Support Organization

[56] Horvatits T, Drolz A, Trauner M, Fuhrmann V (2019) Liver injury and failure in critical illness. Hepatology 70:2204–2215 31215660 10.1002/hep.30824

[57] Kormoczi GF, Saemann MD, Buchta C et al (2006) Influence of clinical factors on the haemolysis marker haptoglobin. Eur J Clin Invest 36:202–209 16506966 10.1111/j.1365-2362.2006.01617.x

[58] Schaer DJ, Buehler PW, Alayash AI, Belcher JD, Vercellotti GM (2013) Hemolysis and free hemoglobin revisited: exploring hemoglobin and hemin scavengers as a novel class of therapeutic proteins. Blood 121:1276–1284 23264591 10.1182/blood-2012-11-451229 PMC3578950

[59] Carter K, Worwood M (2007) Haptoglobin: a review of the major allele frequencies worldwide and their association with diseases. Int J Lab Hematol 29:92–110 17474882 10.1111/j.1751-553X.2007.00898.x

[60] Shih AWY, McFarlane A, Verhovsek M (2014) Haptoglobin testing in hemolysis: measurement and interpretation: haptoglobin testing in hemolysis. Am J Hematol 89:443–447 24809098 10.1002/ajh.23623

[61] Evans L, Rhodes A, Alhazzani W et al (2021) Surviving sepsis campaign: international guidelines for management of sepsis and septic shock 2021. Intensive Care Med 47:1181–1247 34599691 10.1007/s00134-021-06506-y PMC8486643

