# Supplementary material for: Hemolysis, hemolytic markers, and mortality in sepsis: a scoping review
Source: Intensive Care Med Exp. 2025 Aug 5;13:79. doi: 10.1186/s40635-025-00786-0 (PMC12325840; doi:10.1186/s40635-025-00786-0)
Supplement: Supplementary file 1 — Additional file 1. [file 40635_2025_786_MOESM1_ESM.pdf]

Additional File 1

Hemolysis, hemolytic markers and mortality in sepsis – a scoping review

Authors

Victoria Bünger[1,2], Stephanie Scholz[1], Martin Russ[1,2], Steffen Weber-Carstens[1,2], Jan A. Graw[1,3]

1 Search Strategy

Pubmed:

(plasma haemoglobin OR plasma hemoglobin OR cell-free haemoglobin OR cell-free hemoglobin OR free haemoglobin OR free hemoglobin OR haptoglobin OR haemolysis OR hemolysis) AND (Sepsis OR Septic shock OR Bacteremia OR Fungemia OR Septicemia OR Systemic inflammatory response syndrome OR SIRS)

EMBASE OVID:

((plasma haemoglobin or plasma hemoglobin or cell-free haemoglobin or cell-free hemoglobin or free haemoglobin or free hemoglobin or haptoglobin or haemolysis or hemolysis) and (Sepsis or Septic shock or Bacteremia or Fungemia or Septicemia or Systemic inflammatory response syndrome or SIRS)).ti,ab.

Web of Science:

(plasma haemoglobin OR plasma hemoglobin OR cell-free haemoglobin OR cell-free hemoglobin OR free haemoglobin OR free hemoglobin OR haptoglobin OR haemolysis OR hemolysis) AND (Sepsis OR Septic shock OR Bacteremia OR Fungemia OR Septicemia OR Systemic inflammatory response syndrome OR SIRS)

2 Supplemental Tables

Table S1: Etiology and focus of sepsis

Author Year	Etiology of sepsis
Adamzik 2012	n/a
Boshuizen 2019	n/a
Caya 1986	n/a
Czempik 2023	respiratory 36%<br>abdominal 29%<br>urinary 23%
Domizi 2024	respiratory 56%<br>genitourinary 20%
Ekregbesi 2018	respiratory 65.7%<br>soft tissue 12.9%<br>intraabdominal 11.4%<br>bladder 8.6%<br>bone 1.4%
Englert 2019	respiratory 44.4%<br>soft tissue 16.7%<br>abdominal 27.8%<br>endocarditis 5.6%<br>catheter-related 5.6%
Fujita 2010	n/a
Gaini 2007	respiratory 55.4%<br>other 18.9%<br>urinary 12.2%<br>endocarditis 1.4%<br>abdominal 1.4%<br>soft tissue 1.4%<br>bone 1.4%
Gaini 2008	urinary 34.9%<br>respiratory 23.3%<br>other 23.3%<br>endocarditis 5.8%<br>soft tissue 4.7%<br>bone/joint 4.7%<br>meningitis 3.5%
Han 2015	blood 45%<br>pulmonary 32%<br>urinary 10%<br>SBP 7%<br>mastoiditis 3%<br>C. Diff 3%
Janz_1 2013	n/a
Janz_2 2013	n/a
Janz 2015	respiratory 47.5%<br>urinary 30%<br>abdominal 17.5%<br>vascular 17.5%<br>skin 5%

	CNS 2.5%
Kelly 2018	medical patients 50.4%<br>surgical patients 49.6%
Kerchberger 2019	n/a
Kingston 2020	n/a
Lan 2022	n/a
Larsen 2010	surgical 71%
Leff 1992	n/a
Maiden 2018	blood 8.6%
Memis 2002	pneumonia 83.3%<br>peritonitis 16.7%
Mizuno 2022	abdominal 41.0%<br>others 20.5%<br>soft tissue 10.3%<br>respiratory 2.6%
Nilsson 2020	airway infection 26.6%<br>nosocomial infection 9.5%
Reah 1997	abdominal 78.6%<br>urinary 7.1%<br>respiratory 14.3%
Sharma 2019	respiratory 100%
Shindo 2015	abdominal 45%
Spies 1994	n/a
Staudinger 1996	respiratory 63%<br>urinary 4.2%<br>cns 8.3%<br>abdominal 8.3%<br>haema/onco 16.7%
Sun 2024	respiratory 46.6%<br>urogenital 16.5%<br>digestive 15.4%<br>other 16.2%<br>cardiovascular 5.3%
Suzaki 2021	abdominal 54.5%<br>respiratory 18.2%<br>trauma 9.1%<br>genital 9.1%<br>unknown 9.1%
Talluto 1975	n/a
Tanaka 2024	abdominal 44.5%<br>lung 23.0%<br>skin/tissue 12.7%<br>urinary 4.8%<br>blood 3.9%<br>others 10.9%
Wynne 1971	Abdominal 76.9%

Table S2: Association of hemolytic markers with mortality and secondary outcomes

Study year	Impact on mortality	Impact on secondary outcomes
Adamzik 2012	ELISA OR 2.33 (95%CI 1.17-4.65); p=0.015<br>Harboe OR 6.06 (95%CI 2.79-7.80); p< 0.001<br>Noe OR 5.21 (95%CI 2.44-11.11); p< 0.001<br>Fairbanks OR 3.92 (95%CI 1.90-8.06); p<0.001	n/a
Boshuizen 2019	Unclear	n/a
Caya 1986	Mortality with hemolysis: 100% (n=8), p=0.03**	n/a
Czempik 2023	Unclear/Bili not provided for survivors and non-survivors	n/a
Domizi 2024	Unclear	SOFA with CFH_{2h}:<br>Spearman's correlation coefficient 0.306, p=0.032
Ekregbesi 2018	HR [95%CI] over/under median:<br>Heme: 4.04 [1.29-12.67], p=0.016<br>HPX: 0.53 [0.20-1.45], p=0.219	Correlation of APACHE II and<br>Hp: r=0.14, p=0.2514<br>Heme: r=0.1637, p=0.1757<br>HPX: r=0.0251, p=0.8366
Englert 2019	median heme*<br>survived: 6 µM<br>deceased: 7.5µM, ns	SOFA (day 1) vs Heme/HSA p= 0.827
Fujita 2010	Mortality with hemolysis: 100% (n=2) vs. mortality without hemolysis: 18.8% (n=16), p= 0.065**	n/a
Gaini 2007	Unclear	n/a
Gaini 2008	Unclear	n/a
Han 2015	Unclear	n/a
Janz 1 2013	medianCFH at enrollment:<br>Survivors: 10 (10-30) mg/dl vs. non-survivors: 20 (10-40) mg/dl; p=0.002<br>Logistic regression: OR 1.078 [1.012-1.149]; p=0.02 per mg/dl CFH<br><br>CFH:<br>Mortality of patients with undetectable CFH (<10mg/dL): 6/75 (8.1%) vs. patients with detectable CFH (10-70mg/dL): 79/316 (24.9%), p≤ 0.001	n/a
Janz 2 2013	medianHp:<br>Survivors: 1234µg/ml (569-3037) vs. non-survivors: 750µg/ml, (404-2421); p=0.008<br><br>medianHPX:<br>Survivors: 616µg/ml, (397-934) vs. non-survivors: 470µg/ml, (303-891); p=0.012<br><br>CFH:<br>Mortality of patients with detectable (10-80mg/dl) cell-free hemoglobin (n=310): 25.2% vs. patients with no detectable cell-free hemoglobin (n=77): 7.8% (P <0.001)<br><br>Univariate logistic regression:<br>Hp OR 0.589, [0.399-0.87]; p=0.007<br>HPX OR 0.241, [0.098-0.596]; p=0.002<br><br>Multivariate regression Hp:	n/a

	CFH (log) OR 2.152 [1.324-3.497]; p=0.002<br>Hp (log) OR 0.653 [0.433-0.984]; p=0.042<br><br>Multivariate regression HPX:<br>CFH (log) OR 2.143 [1.323-3.471]; p=0.002<br>HPX (log) OR 0.530 [0.199-1.416]; p=0.206<br><br>Patients without elevated CFH (n=77):<br>Hp OR 0.751, [0.168-3.364]; p=0.737<br>HPX OR 2.762, [0.062-122.805], p=0.584	
Janz 2015	Unclear	n/a
Kelly 2018	Hp (mg/dl) and 14d-mortality (Survivors vs. Non-Survivors):<br>baseline: 99.78 (119.43) vs. 99.78 (70.83)<br>24h: 99.78 (86.3) vs. 99.78 (81.59)<br>48h: 99.78 (148.03) vs. 52.43 (147.61)<br>72h: 99.78 (182.52) vs. 67.78 (129.93), all p values not significant<br><br>Hp (mg/dl) and In-Hospital-mortality:<br>baseline: 99.78 (143.03) vs. 99.78 (69.45)<br>24h: 99.78 (96.84) vs. 99.78 (73.33)<br>48h: 99.78 (201.28) vs. 99.78 (125.47)<br>72h: 99.78 (181.43) vs. 99.78 (140.03), all p values not significant<br><br>AUROC:<br>For 14d-mortality<br>baseline 0.563<br>24h 0.580<br>48h 0.619<br>72h 0.591<br><br>For In-Hospital mortality<br>baseline 0.540<br>24h 0.554<br>48h 0.565<br>72h 0.538	n/a
Kerchberger 2019	Mortality for Hp-genotypes<br>HP1-1: 15 (20%)<br>HP2-1: 49 (22%)<br>HP 2-2: 42 (21%)<br>P= 0.925	Development of ARDS (OR)<br>CFH per 50mg/dl: 1.12 (0.91, 1.37); p=0.29<br><br>per CFH quartiles (p=0.032):<br>undetectable (n=82): 28%<br>10-19mg/dl (n=130): 35.4%<br>20-39mg/dl (n=156): 38.5%<br>≥40mg/dl (n=128): 40.6%
Kingston 2020	Unclear	n/a
Lan 2022	Mortality for iHp (<95 mg/dL, n=165) vs. (95-215 mg/dL, n=169) vs. (>215 mg/dL, n=167)<br><br>28-day (p=0.018):<br>88 (53.3%)/70 (41.4%)/65 (38.9%)<br><br>90-day (p=0.020):<br>102 (61.8%)/88 (52.1%)/78 (46.7%)<br><br>ICU (p=0.002):<br>84 (50.9%)/57 (33.7%)/58 (34.7%)	Association iHp with SOFA, p<0.001<br>median iHp Values*:<br>SOFA <7: 205 (120-305) mg/dl<br>SOFA 7-9: 160 (80-165) mg/dl<br>SOFA 9-12: 150 (50-120) mg/dl<br>SOFA>12: 105 (40-100) mg/dl

	Hospital (p=0.003):<br>93 (56.4%)/68 (40.2%)/67 (40.1%)<br><br>28-day mortality Cox regression for iHp value<br><95 mg/dL Reference (OR 1)<br>95-215 mg/dL HR 0.737 [0.527-1.001], p 0.051<br>>215 mg/dL HR 0.653 [0.468-0.910], p= 0.012	
Larsen 2010	28d-mortality:<br>median $HPX_{non-survivors}$*: 0.75 (0.6-1.0) mg/ml<br>median $HPX_{survivors}$*: 1.25 (0.9-1.3) mg/ml; p<0.005<br>Survival time ~HPX concentration p<0.005	HPX and SOFA (survivors) and non-survivors<br>day 1 -0.33 (P=0.26) -0.41 (P=0.08)<br>day 2 -0.40 (P=0.20) -0.13 (P=0.61)<br>day 3 -0.10 (P=0.75) -0.58 (P=0.048)<br>day 5 -0.63 (P=0.051) -0.93 (P=0.002)<br>day 7 -0.63 (P=0.051) -0.58 (P=0.08)
Leff 1992	Unclear	$mBili_{ARDS}$ 0.6 ± 0.1mg/dl vs. $mBili_{NoARDS}$ 2.6 ± 1mg/dl, p>0.05<br>mHp_{ARDS} 220 mg/dl* vs. mHp_{NoARDS} 225 mg/dl*, p>0.05
Maiden 2018	Unclear	n/a
Memis 2002	Unclear	n/a
Mizuno 2022	No difference in 180-day mortality between low Hp and high Hp groups<br>BUT: in high HMGB1 Group: High HP: OR 0.131 [0.027–0.629], P=0.011<br>Multivariate analysis with SOFA score: adj. OR 0.086 [0.013–0.582], P=0.009	n/a
Nilsson 2020	Unclear	n/a
Reah 1997	Unclear	n/a
Sharma 2019	no significant difference in Hp between survivors and non-survivors	n/a
Shindo 2015	4/21 dead, 1 patient with hemolysis, p=0.1905**	n/a
Spies 1994	Unclear	n/a
Staudinger 1996	Unclear	n/a
Sun 2024	Unclear	n/a
Suzuki 2021	$Hemolysis_{Surv}$: 0%<br>$Hemolysis_{Non-Surv}$: 83.33%, p=0.015**	n/a
Talluto 1975	1 Patient with hemolysis: deceased, ns, p=0.1**	n/a
Tanaka 2024	Unclear	n/a
Wynne 1971	Unclear	n/a
*values approximated from figure, **p values calculated with Fisher´s exact test.		
